# Development of Obstetric Practice During the Early Islamic Era

**DOI:** 10.1007/s43032-022-00887-1

**Published:** 2022-03-01

**Authors:** Hossam E. Fadel, Ayman Al-Hendy

**Affiliations:** 1 Present Address: Maternal Fetal Medicine, Department of Obstetrics and Gynecology, (retired), The Medical College of Georgia, Augusta University, Augusta, GA USA; 2 Department of Obstetrics and Gynecology, University of Chicago, Chicago, IL USA

**Keywords:** Islamic civilization, Medieval medicine, Obstetric practice, History, al-Majusi, al-Razi, al-Zahrawi, Ibn Sina, al-Baladi, Ibn Maimon

## Abstract

Art of healing was considered the most noble of human undertakings by Islamic scholars. Acquiring medical manuscripts from previous civilizations and translating them into Arabic proceeded at a great pace. This was followed by the emergence of several great physician scientists who examined these writings, corrected many, and proceeded to produce their own, with the addition of significant original paradigm-shifting contributions to all branches of science and medicine. This article highlights some of the most important contributions to obstetrics of several prominent scholars of the early Islamic period (700–1300 A.D.)

## Introduction

The Islamic golden age spanned almost seven centuries beginning in the seventh century C.E. Islam stresses the importance of seeking knowledge. No wonder Muslims early on sought knowledge in all its spheres, probably most enthusiastically in the art of healing. This was inspired by a *hadith* (a saying attributed to Prophet Muhammad):

Allah (God) has sent down both the disease and the cure, and He has appointed a cure for every disease, so treat yourselves [1].

This movement started by a determined effort by several scholars and the support of enlightened rulers to acquire all knowledge available at the time. Translations from Greek, Syriac, Sanskrit, Persian, and Egyptian manuscripts proceeded at a high pace in the eight and ninth centuries C.E. Translations of Hippocrates, Aristotle, and Galen books into Arabic became available [2, 3]. Many prominent Muslim physicians wrote books correcting prior concepts and added their own observations based on actual clinical practice and experimentation. Among these prominent physicians are al-Razi (Rhazes, 841–926 CE), al-Zahrawi (Albucasis, 930–1013 CE), al-Majusi (Haly Abbas, died 994 CE), ibn Sina (Avicenna, 980–1037 CE), al-Baladi (early eleventh CE), and ibn Maimon (Maimonides, 1135–1204 CE). Their books were translated into several languages, including Latin, and were used for teaching in European medical schools up to the eighteenth century [4–6].

This article is limited to contributions to the field of obstetrics.

## Diseases of Women as a Separate Branch of Medicine

While it is possible that ancient civilizations in Egypt, the Fertile Crescent, India, and China had separate specialties in medicine, it is the Muslims who made midwifery and diseases of women a special branch of the medical arts. In contrast, gynecology was separated from the field of surgery in European medicine only in the early nineteenth century [7].

## Women in Medicine in the Early Islamic Era

Women were actively involved in the practice of medicine, especially labor and delivery They were called “dayas” (midwives). They mostly worked under the supervision of male physicians, but many were independent. Ibn Zuhr, known in the West as Avenzoar (1094–1161), was one of the most renowned physicians of Ishbiliyyah (Seville) in Andalusia. His daughter and granddaughter were the first known female obstetricians [7]. Darwish and Weber reported that many women healers were able to pursue careers in medical institutions with established salaried positions both in Egypt and in the Ottoman society at large. They report on the presence of illustrations of a woman performing gynecologic surgery and another woman extracting a hydrocephalic dead fetus in the thirteenth century [8].

## Examples of Contributions to Obstetrics in the Early Islamic Period

Al-Majusi in his book *Kamil al-Sina’ah al-Tibbiyyah (*The complete Medical Profession), was the first to describe uterine contractions as the cause of delivery. Before that, it was thought that contractions are only the indication of onset of labor but subsequently the fetus swims its way out of the womb [4]. Hippocrates likened delivery to the process by which the chick hatches out of the egg [9].

Al-Razi devoted a whole chapter (third volume, part 9) of his book *al-Hawi fil-Tibb* (The Comprehensive Book), known in the West as *Liber Continens*, to diseases of pregnancy, labor and delivery [10]. Al-Razi, made the following relevant statements:

- Fetuses born before the eighth month commonly present with the breech and usually die.
- The fetus changes its position from breech to vertex in the eighth month because the head is the heaviest part of the body and will gravitate downwards.
- “If the foot or the hand presents, it can cause death of the fetus and mother.” This probably is a reference to transverse presentations, which leads to obstructed labor and eventually to ruptured uterus.
- “Twins are delivered within few days of each other at the most.”
- “Sexual intercourse brings on labor and facilitates delivery.” This observation is now explained by deposition of seminal oxytocic prostaglandins in the vagina.

Al-Razi correctly noted that acute fever of the mother can cause fetal death. He also noted that “if the breast undergoes regressive changes, the fetus will die.” Breast regression during pregnancy can be understood nowadays to be the result of low levels of prolactin and progesterone, secondary to severe placental insufficiency that can also be associated with fetal death. He also noted that if labor pains are in the pelvic area, labor will usually be easy, but, if the pain is mostly in the lower back, labor will usually be difficult (referring to the occipito-posterior position of the fetal head).

Al-Razi instructed midwives to examine the parturient before embarking on the delivery. Specifically, the cervix needs to be checked to see how much it is dilated, to determine what is the presenting part, and to follow the progress of cervical dilation until it is sufficiently dilated. Then they can ask the parturient to push down the fetus.

AI-Razi described the complication of umbilical cord around the neck and stated that it can be a cause of difficult labor and fetal death. AI-Razi gave different prescriptions of medicinal herbs with their respective dosages and recommended special kinds of food to “facilitate" labor and delivery.

He described the operation of internal podalic version and gave a brief description of the destructive operations to deliver a dead fetus.

Al-Zahrawi, was born in 936 C.E.at *al-Zahra'* near *Qurtobah* (Cordova), Spain. His greatest contributions are contained in his famous book *al-Tasrif* which is known in the West as *Chirurgia*. *Al-Tasrif* consists of 30 volumes each consisting of several chapters. Chapters 74–78 are devoted to Obstetrics and Gynecology. It was translated into Persian, Hebrew, and Latin. The Latin translation was by Gerard of Cremora and dates to the last half of the twelfth century CE. A recent translation to English was rendered by Spink [11] and more recently by Spink and Lewis [12]. Al-Zahrawi gave detailed account of all possible malpresentations: prolapsed hand, incomplete breech, complete breech, transverse presentation, and cord around the neck. He then detailed maneuvers to affect delivery under such circumstances such as replacing the hand, internal podalic version. In difficult vertex deliveries, he would resort to changing the position of the parturient, shaking her, placing her in a special seat, Valsalva maneuver, etc. If unsuccessful, he would recommend mixing mucilage of fenugreek, oil of fumary, and gum and pounding them in a mortar and then anointing the woman’s perineum and make her sit down in warm water reaching to the ribs. Then, he would make a suppository of murrh and introduce it in the vagina and after an hour make the woman stand [12].

Al-Zahrawi recognized multifetal pregnancy. He correctly noted that twins are commonly born alive; triplets or quadruplets can sometimes be born alive and higher order multifetal pregnancies are usually aborted early in the pregnancy. He noted that twins can be in one sac (monochorionic) or in separate sacs (dichorionic).

**Fig. 1:**
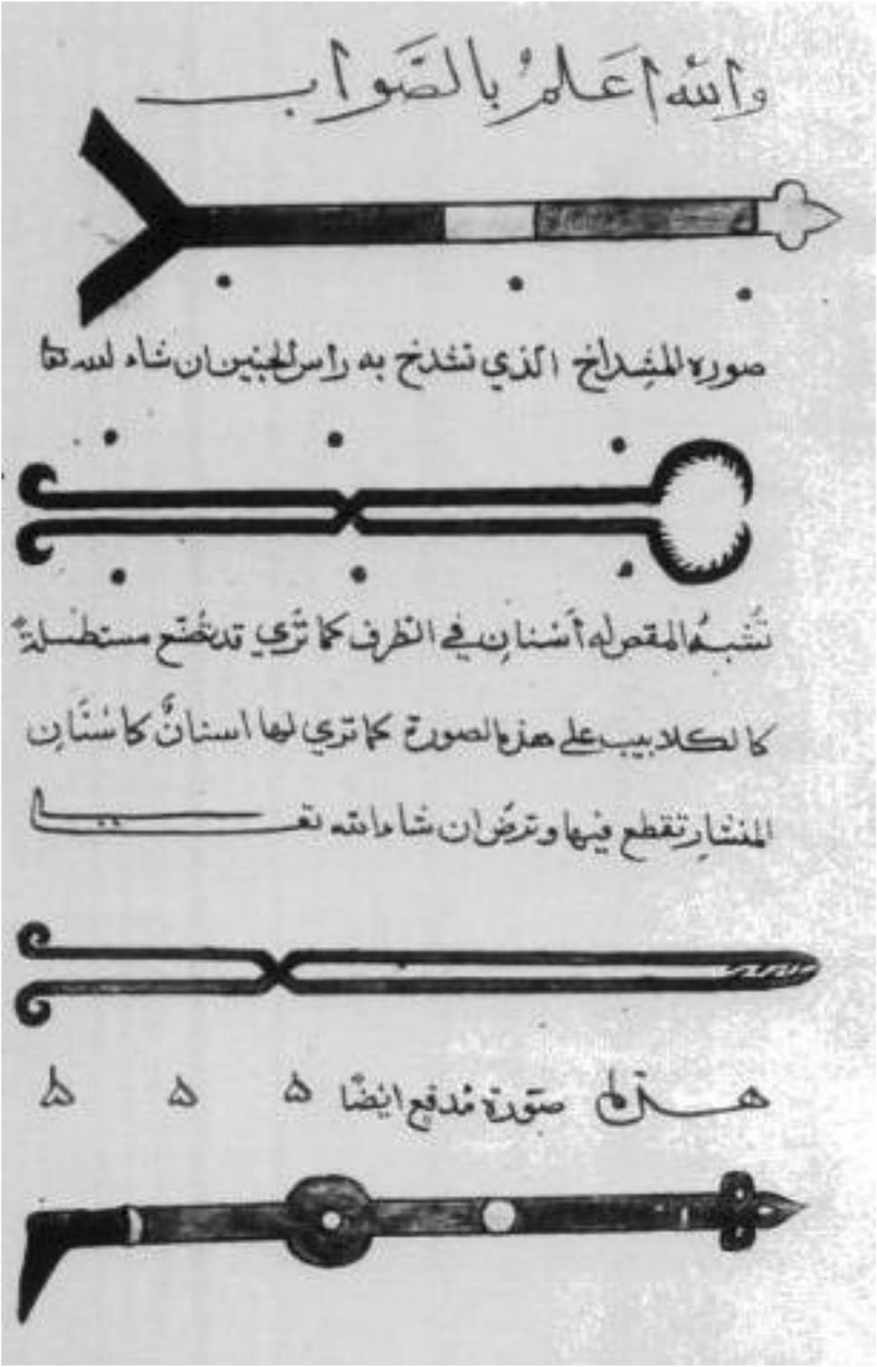
Illustration of medieval Muslim surgical instruments taken from al-Zahrawi’s Kitab al-Tasrif. Fifteenth century copy of an eleventh century manuscript. The top instrument is explicitly stated to be for perforating the fetal head (craniotomy). Probably the bottom instrument is used for the same. The other two instruments probably can be used for evisceration (embryotomy). Additional related figures can be found in reference No. 13. Attribution and permission: https://en.wikipedia.org/wiki/File:Zahrawi1.png#/media/File:Zahrawi1.png

AI-Zahrawi described more than 200 surgical instruments, almost all of which were illustrated in his book *al-Tasrif*. Many were of his own design [6]. Many of these were to be used in gynecological examinations and treatment, to facilitate delivery, and or to deliver and extract dead fetuses. These illustrations probably formed the basis of the design for some modern obstetric instruments (Fig. 1). Additional related figures have been previously published [13]. These include *midfaa* (thruster), perforator (craniotomy scissors, cranioclast), *mibdaa’* (scalpel), *mishdakh* (crucher, cephalotribe), *sinnarah* (hook, crotchet), *miqass* (scissors), and *kalalib* (claws) [12].

His description of the operation to extract a dead fetus shows his thoroughness and the effectiveness of his instructions to midwives. It was fully and accurately translated by Spink and Lewis [12].

Al-Zahrawi also described the operation of craniotomy (and other destructive operations) in detail with extraordinary degree of knowledge and experience.

If the fetal head is large, and it is tightly squeezed in exit, or if there is a collection of fluid in the head (hydrocephalus), you should introduce between your fingers a spike shaped scalpel (perforator) and split the head to let the water out or you should smash it with the instrument called *mishdakh* (crusher, the modern cranioclast or cephalotribe), then you should draw out the bones with the forceps. If the head comes out and the fetus is held up at the collar bones (shoulder dystocia), an incision should be made (cliedotomy). If the thorax is impacted, perforate to let out the humidity in it (hydrothorax), the thorax will then shrink. But if it does not, then you cut off pieces in any manner possible (evisceration). If the lower belly is swollen or dropsical (ascites) then you should make an opening to draw out all the fluid. If the fetus presents by the feet, then the extraction will be easy, and it will be a simple matter to guide it to the maternal opening. If it is stuck about the thorax or abdomen, then pull on it with a cloth around your hand and cut an opening in the abdomen or thorax to allow the contents to flow out (evisceration). If the fetus presents laterally (transverse) and it is possible to reposition it (podalic version) apply the maneuvers for a living fetus, but if this is not possible then the fetus should be cut away piecemeal, then extracted [12].

AI-Zahrawi stressed that the placenta should be removed completely after delivery. “It is necessary that not a scrap of the afterbirth be left behind in the womb.” He described methods to affect placental delivery. First, let the woman sneeze while closing her mouth and nose. Then, use vapors of certain herbs introduced in the uterus while the woman is sitting. If this fails, he describes manual removal. He stresses the importance of separating the placenta from the uterine wall gently and then pulling it from side to side, avoiding violent pulling that can result in rupture of the uterus or *Inqlab al-Rahim* (uterine inversion). He realized that sometimes removal fails (placenta accreta) and that one has to stop these maneuvers, probably first mention of this condition. In this circumstance, he injects tetrapharmacon ointment in the uterus that will cause putrefaction of the placenta in a few days. That will loosen it and it will come out [12].

AI-Zahrawi described a case of abdominal pregnancy that was mistaken for a case of intra uterine fetal death (IUFD). The extrauterine sac turned into an abscess, which started drainage with extrusion of the bones of the dead fetus. With proper treatment (evacuation and dressing), the woman survived for a long time. A translation of his full description of the case is cited [12].

Ibn Sina’s most famous book, *al- Qanun fil-tibb*, was known in Europe as *The Canon*. He was born in Bukhara in present-day Uzbekistan and died in Isfahan in present-day Iran [4–6]. The *Qanun* had several articles on obstetrics [14]. He stressed the importance of healthy lifestyle of both parents to ensure healthy offspring. He is probably the first to indicate the importance of the father’s health in this regard. He mentioned 6 principles of healthy lifestyle: exercise, nutrition, sleep and awareness, excretion of body wastes, psychic features as well as air and climate. These are as pertinent now as when they were stated by ibn Sina 1000 years ago [15]. Another accurate statement by ibn Sina is that “delivery occurs when the fetus cannot get enough blood from the placenta.” We now know that placental functions decline as term approaches and that may trigger the initiation of labor. If delivery is delayed (post-dates), placental insufficiency increases and may cause fetal death [14].

Ibn Sina classified the causes of abnormal labor (dystocia) into maternal, fetal, faults in the uterus or placenta, timing of delivery (preterm or post-term), or mistakes by the midwife.

He enumerated maternal causes: the parturient may be weak, suffering from diseases, undernourished, a primigravida, too scared, elderly, obese, unable to squeeze the uterus with her abdominal muscles, restless such that she rolls from one side to the other frequently or impatient with the labor pains. Other causes include tumors of the bladder, urinary retention, tumors of the rectum and colon, and impacted hard fecal matter.

Fetal causes include female gender, big size, big head, being too small (light) such that it cannot forcibly fall down and some anomaly such as double head. Multifetal pregnancy leads to abnormal labor. A dead fetus cannot help in the process. A malpresenting fetus (legs or knees;breech, or by side; transverse, or with hands, i.e., prolapsed arm) is a fetal cause for dystocia.

Uterine causes include small size (probably referring to a contracted pelvis), improperly healed cervical tear or “hemorrhoids’ of the uterus. Placental causes are thick placenta (placenta previa), premature rupture of membranes and “dry” uterus (oligohydramnios) such that the birth canal is not slippery. Amazingly, this classification nearly mirrors our current one.

Ibn Sina also discussed the management of difficult labor. For example, he described how to deliver a fetus that is coming by *janb* (side), i.e., transverse presentation, first by manipulation and, if unsuccessful, by the use of *kalalib* (hooks) and, if unsuccessful, by dividing it in pieces (evisceration) as in the delivery of a dead fetus. He also described difficult labor: if the “head bone is big,” “open it up so the inside liquid flows out (probably describing craniotomy for hydrocephalus).”

He discussed the management of a dead fetus or the one that there is no hope of being born alive, that in case of “labor lasting for more than 4 days the fetus must be dead.” In these cases, ibn Sina would use ointments and grab the fetus manipulating it to be de extracted. If unsuccessful, he advised attaching hooks and cutting the fetus in pieces (evisceration). He further advised to try to effect delivery quickly, otherwise “the dead fetus will swell (rot) and its extraction will become more difficult.” [14]

To effect delivery of the placenta, ibn Sina recommended that the parturient sneeze or blow out with her mouth closed while holding her nostrils. He described manual removal of the placenta accurately, stressing the importance of gentle pulling to avoid inversion. If this fails, he prescribed medications and vapors.

Dunn reported that ibn Sina was the first to associate prolonged and difficult labor with intractable and persistent urinary incontinence (vesico-vaginal fistula) [16]. Dunn also stated that “with regard to the fillet and forceps, they have been alleged to be late inventions; yet we find Avicenna recommending the use of both.” [16]

Ibn Sina discussed the outcome of preterm delivery. Fetuses delivered before the seventh month are too weak to survive. He mistakenly reported, as did Hippocrates before him, that fetuses born at the eighth month are weak and more prone to die than those delivered at the seventh month. If fetuses remain in utero until the ninth month, they will “recover” and become stronger and will survive when born.

Ibn Sina discussed the causes of fetal and infant deformity:

Some of these agents come into play from the beginning because of a defect in the formative power of the sperm (genetic factors). Others come into force later in life, namely in parturition, during the act of traversing the maternal passages. Others operate after birth (tight binders and wrappings). Others operate in infancy, before the limbs are hard enough to enable the infant to walk [14].

Prenatal care seems to be first dealt with as a separate entity by al-Baladi in his book titled *Tadbir al-hawamil* (Management of pregnant women, children, boys and the management of their diseases). He was born in a city near Mosul in present-day Iraq, probably in the early part of the fourth Hijri century (tenth CE) [17].

He correctly observed that fetal health/wellbeing depends on the mother’s health. Active women with healthy bodies, few complaints, good spirit, demeanor, digestion, and temperament who move easily are prone to have strong fetuses in good health. He stated that having milk secretion from the breasts before delivery indicates weakness of the fetus. Pregnant women need more nutrition but not too much, as this may disturb their stomach. The increase should be gradual and consist of easily digested food. In concordance with the increase in diet, there should be an increase in exercise and movement. She should avoid jumping, carrying heavy loads and stooping down, avoid loud noises (sound of thunder), and traumatic events which can cause miscarriage.

He discussed sexual activity during pregnancy. Women should not completely abstain from sex during pregnancy as complete abstinence is not good for the pregnant woman. It will cause difficult labor and make labor pains stronger. It is better to avoid it in the first 2 months and after the sixth month. At the latter time the fetus is heavy and cannot be trusted to fall during intercourse because of the excessive movement and the fetus is already ready to get out. But also “too frequent” sex is not good. It can weaken the fetus and possibly be a cause for miscarriage. Obstetricians today continue to recommend to women with a history of abortion to abstain from sex in the first trimester and to those with a history of preterm labor to abstain in the last trimester.

Al-Baladi states that pregnant women usually have stomach upsets, nausea, and vomiting. He recommended managing these symptoms with different food items and herbals. He discussed *waham* (craving) and increased salivation (sialorrhea) [17]. These are common ailments of pregnancy we frequently encounter in our patients.

Musa ibn Maimon (Maimonides, 1135–1204 CE), the prominent Jewish theologian, philosopher, and physician, was born in Qurtobah (Cordova, Andalusia). He ended up as the chief physician in Sultan Saladin’s court in Cairo, Egypt, where he died. Chapter 16 of his book *Medical Aphorisms* is titled “Doctrines Pertaining to Female Disorders.” [18] He described craving for food (pica). He theorized it to be due to “bad juices in the folds of the stomach.” Pica subsides at the fourth month because these “bad juices will be spit through vomiting.” He stated that “shriveled breasts forebode abortion. If pregnant with twins and one of the breasts shrivel, she will abort one of the fetuses. The same has been mentioned in the book *al-Hawi* [10]. Ibn Maimon also noted that the pulse becomes stronger, more rapid and fuller during pregnancy, all correct observations. He also noted that breast milk is the most suitable nutrition for the newborn infant [18].

## Conclusion

Unfortunately, European historians in general have ignored the original contributions of Muslims to the Renaissance. They called the period between Ancient Greek civilization and the Renaissance “The Dark Ages,” ignoring the great civilization that existed during the Islamic golden age.

Only recently have historians started to uncover and report the great scientific contributions of Islamic civilization [19–21].

We like to conclude by quoting Spink:

Attention is specially drawn to the gynecological and obstetrical instruments used by the Arabian doctors. It is shown that, in this branch at least, the Arabians were by no means wholly dependent upon the Classical writers … [T]hey altered and improved, out of recognition, the ideas they received from classical sources. … The speculum, the forceps, the lever and the crotchet mark in a special way the original Arab genius. It is also shown that the Arabs had developed a clear practical idea of what is normal, of what varieties of abnormality were to be met with, and by no means least, of prognosis, in obstetrical practice [11].
